# Plasma ghrelin O‐acyltransferase (GOAT) enzyme levels: A novel non‐invasive diagnosis tool for patients with significant prostate cancer

**DOI:** 10.1111/jcmm.13845

**Published:** 2018-09-06

**Authors:** Enrique Gómez‐Gómez, Juan M. Jiménez‐Vacas, Julia Carrasco‐Valiente, Vicente Herrero‐Aguayo, Ana M. Blanca‐Pedregosa, Antonio J. León‐González, José Valero‐Rosa, José L. Fernández‐Rueda, Teresa González‐Serrano, José López‐Miranda, Manuel D. Gahete, Justo P. Castaño, María J. Requena‐Tapia, Raúl M. Luque

**Affiliations:** ^1^ Maimonides Institute for Biomedical Research of Córdoba (IMIBIC) Córdoba Spain; ^2^ Department of Cell Biology, Physiology, and Immunology University of Córdoba Córdoba Spain; ^3^ Reina Sofia University Hospital (HURS) Córdoba Spain; ^4^ Urology service HURS Córdoba Spain; ^5^ CIBER Fisiopatología de la Obesidad y Nutrición (CIBERobn) Córdoba Spain; ^6^ Department of innovation and methodology IMIBIC Córdoba Spain; ^7^ Anatomical Pathology Service HURS Córdoba Spain; ^8^ Lipids and Atherosclerosis Unit HURS Córdoba Spain

**Keywords:** GOAT enzyme, non‐invasive biomarker, significant prostate cancer

## Abstract

Early detection of PCa faces severe limitations as PSA displays poor‐specificity/sensitivity. As we recently demonstrated that plasma ghrelin O‐acyltransferase (GOAT)‐enzyme is significantly elevated in PCa‐patients compared with healthy‐controls, using a limited patients‐cohort, we aimed to further explore the potential of GOAT to improve PCa diagnosis using an ample patients‐cohort (n = 312) and defining subgroups (i.e. significant PCa/metastatic patients, etc.) that could benefit from this biomarker. Plasma GOAT‐levels were evaluated by ELISA in patients with (n = 183) and without (n = 129) PCa. Gleason Score ≥ 7 was considered clinically significant PCa. GOAT‐levels were higher in PCa patients vs control patients, and in those with significant PCa vs non‐significant PCa. GOAT‐levels association with the diagnoses of significant PCa was independent from traditional clinical variables (i.e. PSA/age/DRE). Remarkably, GOAT outperformed PSA in patients with PSA‐levels ranging 3‐20 ng/mL for the significant PCa diagnosis [GOAT‐AUC = 0.612 (0.531‐0.693) vs PSA‐AUC = 0.494 (0.407‐0.580)]. A panel of key variables including GOAT/age/DRE/testosterone also outperformed the same panel but with PSA [AUC = 0.720 (0.710‐0.730) vs AUC = 0.705 (0.695‐0.716), respectively]. Notably, GOAT‐levels could also represent a novel predictive biomarker of aggressiveness, as its levels are positively associated with Gleason Score and the presence of metastasis at the time of diagnoses. Altogether, our data reveal that GOAT‐levels can be used as a non‐invasive biomarker for significant PCa diagnosis in patients at risk of PCa (with PSA: 3‐20 ng/mL).

## 1 INTRODUCTION

Prostate cancer (PCa) has emerged as the most frequent cancer type among men, with an estimation of 164 690 new cases in the United States for 2018 (10% of all new cancer cases).^1^ The rate of diagnosis has increased since the 1990s with the introduction of the PSA test for early detection of PCa, and metastatic disease and specific mortality have been reduced in most western countries.^2^ However, a key limitation in PCa management is that early PCa diagnosis is mainly based on the plasma levels of PSA, a biomarker that exhibits profound drawbacks. For instance, PSA test displays low specificity because of the fact that multiple factors can increase PSA levels, such as benign prostatic hyperplasia or inflammation conditions, and this test is not able to accurately distinguish clinically relevant tumours from indolent cases.^3^ This leads to the overdiagnosis of PCa with many unnecessary biopsies and reduced patient quality of life (QoL), as well as to the overtreatment in a considerable number of patients.^4^ Likewise, clinical management of aggressive PCa, that is metastatic and castration‐resistant PCa (CRPC), also faces major limitations, including unresponsive patients and development of resistance to hormonal and chemical therapies.^5^, ^6^ Therefore, there is an important unmet clinical need for the identification and validation of new, reliable and specific biomarkers for early diagnosis of PCa, as well as for prediction of disease prognosis and treatment response, etc., which would improve patient survival and QoL and would reduce substantially the number of unnecessary biopsies in patients with suspect of PCa based on PSA test.

In line with this, and using a limited cohort of patients, we have recently demonstrated that ghrelin‐O‐acyltransferase (GOAT), a key enzyme regulating ghrelin system activity,^7^, ^8^, ^9^ is overexpressed in PCa tissues (at the mRNA and protein level) and its plasma levels are elevated in PCa patients compared to healthy prostate tissues and to plasma from healthy controls, respectively.^10^ Moreover, we observed that plasma GOAT levels could discriminate PCa, suggesting that GOAT might serve as a potential novel non‐invasive biomarker of PCa.^10^ However, in this previous pilot study, we could not establish whether plasma GOAT levels could be a significantly better diagnostic marker than PSA for the diagnosis of PCa, specially on those individuals with PSA levels ranging 3‐20 ng/mL (wherein precision of PSA is remarkably poor), and for the diagnosis of significant PCa (Sig PCa). Accordingly, the aims of this study were (a) to valorize the utility of plasma GOAT enzyme levels alone, or in combination with other traditional clinical variables, as a tool for the detection of PCa, using a more representative, ample cohort of patients (n = 312) and by defining specific subgroups (e.g. Sig PCa vs non‐Sig PCa) that could benefit from this biomarker; (b) to compare the utility of plasma GOAT vs PSA levels as diagnostic tools in this cohort of patients; and, (c) to determine the utility of plasma GOAT enzyme levels as a novel predictive biomarker of aggressiveness, by analysing its association with Gleason Score (GS), metastatic PCa and earlier CRPC status in the same cohort of patients.

## 2 MATERIAL AND METHODS

This is a case–control study implemented with patients who donated blood under fasting conditions in the Reina Sofia University Hospital. The study was approved by the Hospital Ethic‐Committee, and written informed consent from all patients was obtained. All samples were obtained through the Andalusian‐Biobank (Servicio Andaluz de Salud, Spain).

### 2.1 Patients and samples

Three cohorts of patients were included in the study:

1. Cohort 1: Healthy control population without suspected PCa (65 volunteers with a PSA < 2.5 ng/mL)
2. Cohort 2: Patients at risk of PCa (with suspected PCa) but with a negative biopsy result (64 patients scheduled for prostate biopsies according to clinical practise but with a negative result from the pathology analysis).
3. Cohort 3: Patients at risk of PCa (with suspected PCa) and with a positive biopsy result (183 patients scheduled for prostate biopsies according to clinical practise with a positive result of PCa from the pathology analysis).

Recommendation to undergo prostate biopsies within the population of patients included in this study was: (a) in the case of non‐previous biopsies, suspicious findings on digital rectal examination (DRE), PSA > 10 ng/mL, or PSA 3‐10 ng/mL if free PSA ratio was low (usually, <25%‐30%), and; (b) in patients with previous biopsies (but with a negative result), a persistently suspected PCa. It should be noted that none of the patients was receiving any PCa‐associated medical therapy or was subjected to surgery at the moment of the sample collection. Biopsy specimens were analysed by an uro‐pathologist according to ISUP 2005 modified criteria.^11^

In order to determine and compare the levels of GOAT and PSA in plasma samples from all the patients included in this study (cohorts 1‐3 mentioned above; a total of 312 samples), blood was collected early in the morning, after an overnight fast. Each blood sample was placed into a vacutainer tube containing sodium citrate, centrifuged 10 minutes at 1100 *g* (20°C) and subsequently plasma was aliquoted in tubes and kept at −80°C. Additionally, clinical, anthropometric and pathological features of all the patients were obtained and registered. In addition, testosterone levels were evaluated in patients at risk of PCa (cohorts 2 and 3).

### 2.2 Determination of plasma GOAT, PSA and testosterone levels

For the determination of plasma GOAT levels, a commercial ELISA was used following the manufacture's instructions (MyBioSource, San Diego, USA), as previously reported.^10^ GOAT ELISA kit exhibits a detection limit lower than 0.31 ng/mL and a detection range between 0.78 and 50 ng/mL. The intra‐ and interassay accuracy showed a CV lower than 10% and 12%, respectively. Samples were diluted 1:100 before performing the assay. Levels of PSA and testosterone were measured using technology of Chemiluminescent Microparticle Immunoassays (References 7k70 and 7k73, respectively; Abbott) following the manufacturer's instructions.

### 2.3 Variables and statistical analysis

A descriptive study was performed by calculating the median and interquartile ranges for the quantitative variables and the absolute frequencies and percentages for the qualitative variables. One of the primary endpoints of the study was the presence of a clinically Sig PCa on biopsy. The tumours with a GS ≥ 7 were considered clinically Sig PCa. Student's *t* test was used for analysis of the quantitative data in case of two groups and ANOVA with Bonferroni's post hoc test in case of comparison between the three groups. A chi‐square test was used for the qualitative variables. To study the correlation between GOAT levels and other clinical variables, a Pearson test was used. To address the diagnostic value of both PSA and GOAT measures, their associated ROC curves were built, showing the performance (specificity and sensitivity) for the different risk thresholds. The performance was then compared using DeLong tests over the respective areas under the curves (AUC). Then, the performance of multivariate models based on these measures, when complemented with additional clinical variables (age, DRE, BMI, testosterone, number of biopsies and family history) was investigated. These models were built using logistic regression, preceding the model construction with a feature selection step, using like‐hood ratio test to discard variables that do not contribute to diagnostic performance. The performance of these models was then evaluated using 10‐fold cross‐validation, including the variable selection step to avoid selection bias. Similar to the case of univariate models, ROC curves and DeLong tests were used to compare the different models. An exploratory analysis for the association and prognosis value of GOAT was carried out. For this purpose, data from the follow‐up and treatment with hormonotherapy according to clinical practise were also collected. A univariate Cox Regression analysis was carried out to explore the association of GOAT levels with the development of castration resistant disease (CRPC). A 5% level of significance (after adjusting for multiple comparisons, if specified) was used to decide statistically significant differences to make our conclusions comparable to those of the related research. All the analyses and graphics were performed using GraphPad prism 6, SPSS version 17.0 and R version 3.2.3.

## 3 RESULTS

### 3.1 Descriptive characteristics of the cohort

A total of 312 patients were evaluated (65, 64 and 183 individuals from cohorts 1, 2 and 3, respectively). Clinical characteristics are depicted in Table 1 according to patient category. Patients with PCa (cohort 3) were older compared to patients with negative biopsy (cohort 2) and healthy patients (cohort 1) [67 (62‐72) vs 64 (58‐68) vs 51 (47‐57), respectively; *P* < 0.01]. Patients with PCa had significantly higher plasma PSA levels compared to healthy patients [cohort 3 vs cohort 1; 6.35 (4.15‐12.53) ng/mL vs 0.69 (0.46‐1.03) ng/mL; *P* < 0.05], while a similar, albeit non‐significant trend was found with the patients with negative biopsy [cohort 3 vs cohort 2; 6.35 (4.15‐12.53) ng/mL vs 5.82 (4.42‐6.88) ng/mL; *P* = 0.11]. No differences in BMI between groups of patients were found. The proportion of patients with previous biopsy and normal digital rectal examination (DRE) were significantly higher in cohort 2 (patients with negative biopsy) compared to the group of patients with PCa (*P* < 0.01). Testosterone levels were slightly lower in patients with PCa compared to patients with negative biopsy, but this difference did not reach statistical significance (*P* = 0.09). The percentage of patients with family history did not differ between patients with PCa and with negative biopsy. Finally, 57% of the patients with PCa patients (cohort 3) had a GS of 7 or higher on the biopsy (Sig PCa; n = 105) and 4% (n = 7) presented metastasis at the diagnoses (Table 1).

**Table 1:** Demographic/clinical data and anatomopathological characteristics of the three cohorts of patients included in this study

Variable	Healthy patients	Negative biopsy patients	PCa patients
Patients	65	64	183
Age			
Median (IQR)	51 (47‐57)	64 (58‐68)	67 (62‐72)
PSA level (ng/mL)			
Median (IQR)	0.69 (0.46‐1.03)	5.82 (4.42‐6.88)	6.35 (4.15‐12.53)
BMI			
Median (IQR)	29.07 (26.23‐32.66)	28.23 (26.20‐31.28)	28.44 (25.96‐31.62)
>1 Biopsy		21 (32.8)	27 (14.8)
DRE (Abnormal)	‐	8 (12.5)	69 (37.7)
Testosterone			
Median (IQR)	‐	5.11 (3.99‐6.48)	4.56 (3.69‐5.84)
Family history		10 (15.6)	37 (20.2)
Gleason score			
<7	‐	0	78 (42.6)
≥7	‐	0	105 (57.4)
Metastasis (%)	‐	0	7 (3.8)
Median (IQR) GOAT protein expression	231.68 (189.80‐259.17)	242.42 (211.30‐279.92)	263.51 (220.48‐309.31)

PCa, Prostate Cancer; DRE, Digital Rectal Examination; BMI, Body Mass Index.

Values are expressed in Median (Interquartile range) for quantitative variables and absolute number (Percentage) for qualitative variables.

### 3.2 Capacity of plasma GOAT and PSA levels to predict the presence of PCa and Sig PCa

**Figure 1:**
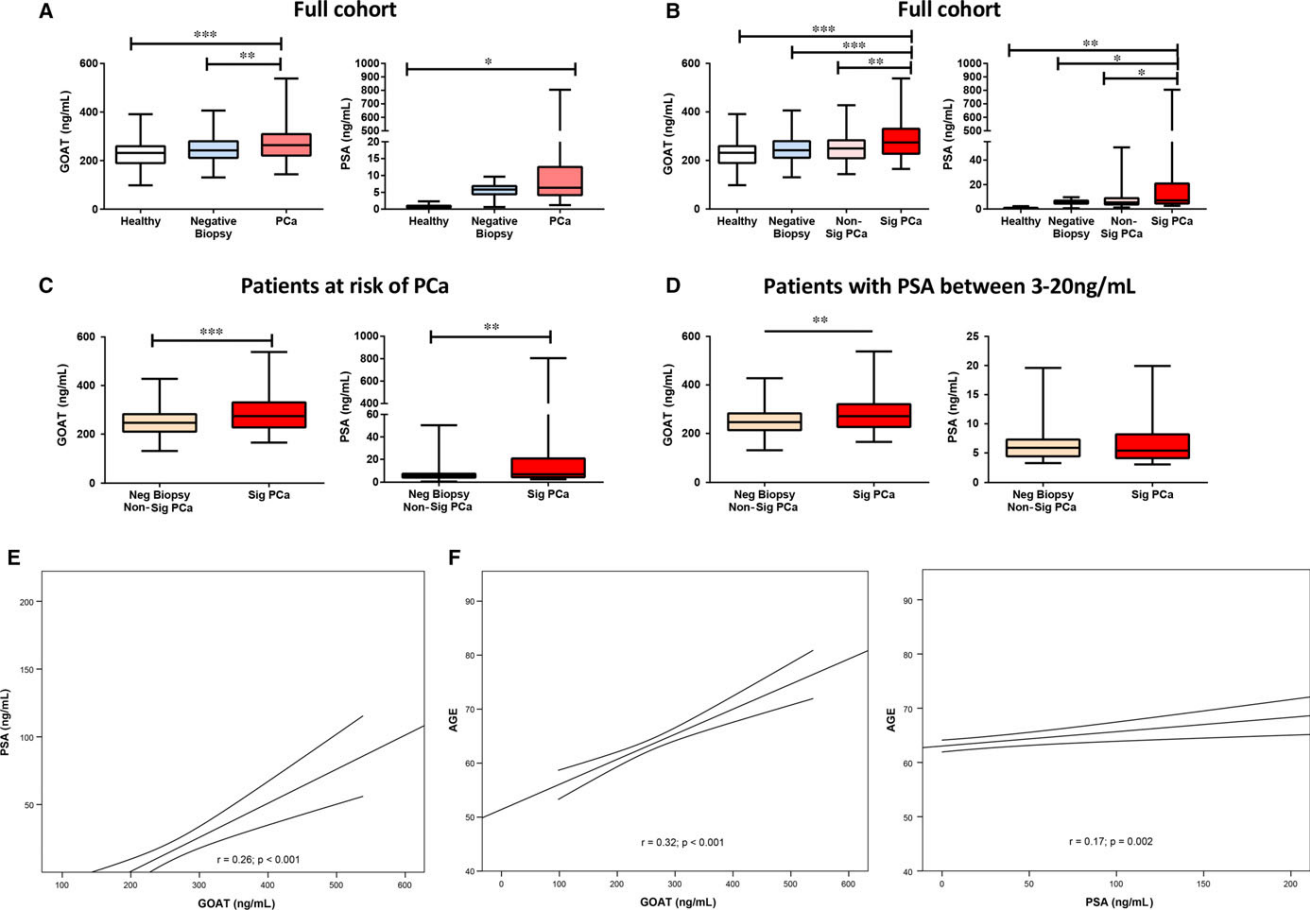
Plasma GOAT and PSA levels according to patient categorization. A, Comparison between plasma GOAT (left‐graph) and PSA (right‐graph) levels in healthy patients (n = 65), patients with suspected prostate cancer (PCa) but with a negative biopsy result (n = 64), and patients with confirmed PCa (n = 183). B, Comparison between plasma GOAT (left‐graph) and PSA (right‐graph) levels in healthy patients, patients with suspected PCa but with a negative biopsy result, and patients with PCa subclassified in non‐significant PCa (non‐Sig PCa; n = 78) and in Sig PCa (n = 105). C, Comparison between plasma GOAT (left‐graph) and PSA (right‐graph) levels in patients with Sig PCa (n = 105) compared to the combined group of patients with suspected PCa but with a negative biopsy together with patients with non‐Sig PCa (n = 142). D, Plasma GOAT (left‐graph) and PSA (right‐graph) levels in patients with Sig PCa compared to the combined group of patients with suspected PCa but with a negative biopsy together with patients with non‐Sig PCa, when considering only the patients with a PSA levels within the 3‐20 ng/mL range (n = 77 and 125, respectively). In all cases, data represent mean ± SEM. Asterisks (*, *P* < 0.05; **, *P* < 0.01, ***, *P* < 0.001) indicate values that significantly differ between groups. E, Correlations between GOAT levels and PSA levels in our cohort of patients. F, Correlations between GOAT (left‐graph) or PSA (right‐graph) levels and age in our cohort of patients. Coefficients of correlation were evaluated by Pearson's test. The graphics show the lineal adjusted method and mean confidence interval

Plasma levels of GOAT were statistically higher in patients with PCa compared to patients with negative biopsy and healthy patients (Figure 1A, left panel). In contrast, PSA levels were higher in patients with PCa compared to healthy patients but not with patients at risk of PCa but with negative biopsy (Figure 1A, right panel). When patients with PCa were divided in two subgroups, with and without Sig PCa, we found that, although both plasma GOAT and PSA levels were significantly elevated in patients with Sig PCa (GS ≥ 7) compared to patients with non‐Sig PCa (GS = 6), these differences were statistically more significant for GOAT vs PSA levels (*P* = 0.002 vs *P* = 0.0145; Figure 1B).

Additionally, plasma GOAT and PSA levels were also found to be higher in patients with Sig PCa compared to the combined group of patients at risk of PCa but with a negative biopsy together with patients with non‐Sig PCa (Figure 1C), being these differences again statistically more significant for GOAT vs PSA levels. Importantly, when the patients with a PSA range between 3 and 20 ng/mL (the most ambiguous region of PSA levels, which leads to a high false‐positive rate and, therefore, to a high number of unnecessary prostate biopsies) were analysed in more detail, we found that plasma GOAT, but not PSA, levels were significantly higher in patients with Sig PCa compared to the combined group of patients with negative biopsy and with non‐Sig PCa (Figure 1D).

Interestingly, plasma GOAT levels positively correlated with plasma PSA levels (Figure 1E), but not with testosterone levels (*r* = −0.044; *P* = 0.49; data not shown), in this cohort of patients, which is consistent with our previous study using a different cohort of patients.^10^ Moreover, a positive correlation was found between plasma GOAT or PSA levels with age (Figure 1F).

### 3.3 Comparison of the predictive ability of GOAT and PSA to detect PCa and Sig PCa in the PSA grey zone

We next applied a multivariate analysis to evaluate the association of plasma GOAT levels with the diagnosis of PCa and Sig PCa adjusting with usual clinical variables analysed in PCa patients (PSA, age, DRE, etc.; Table 2). This revealed that GOAT levels are independent of these variables used in clinical practice, with the strongest association for DRE in the Sig PCa [OR = 4.18 (2.12‐8.24)].

**Table 2:** Multivariate analysis of the association of plasma GOAT levels with the diagnosis of prostate cancer (PCa) and Significant PCa (Sig PCa) adjusting with common clinical variables

Variable	PCa (n = 183)			Sig PCa (GS ≥ 7; n = 105)		
	OR	*P*	95% CI (OR)	OR	*P*	95% CI (OR)
PSA (ng/mL)	1.140	0.010	1.032‐1.259	1.040	0.061	0.998‐1.083
Age	1.043	0.078	0.995‐1.094	1.070	0.003	1.024‐1.119
DRE	2.573	0.031	1.090‐6.074	4.177	0.000	2.118‐8.235
Previous biopsy	0.333	0.004	0.156‐0.710	0.495	0.084	0.223‐1.100
Family history	1.479	0.360	0.640‐3.417	1.104	0.800	0.513‐2.376
GOAT (ng/mL)	1.006	0.049	1.000‐1.012	1.007	0.005	1.002‐1.012

GS, Gleason Score; DRE, Digital rectal examination; Previous Biopsy (Yes vs No); Family History (Yes vs No).

**Figure 2:**
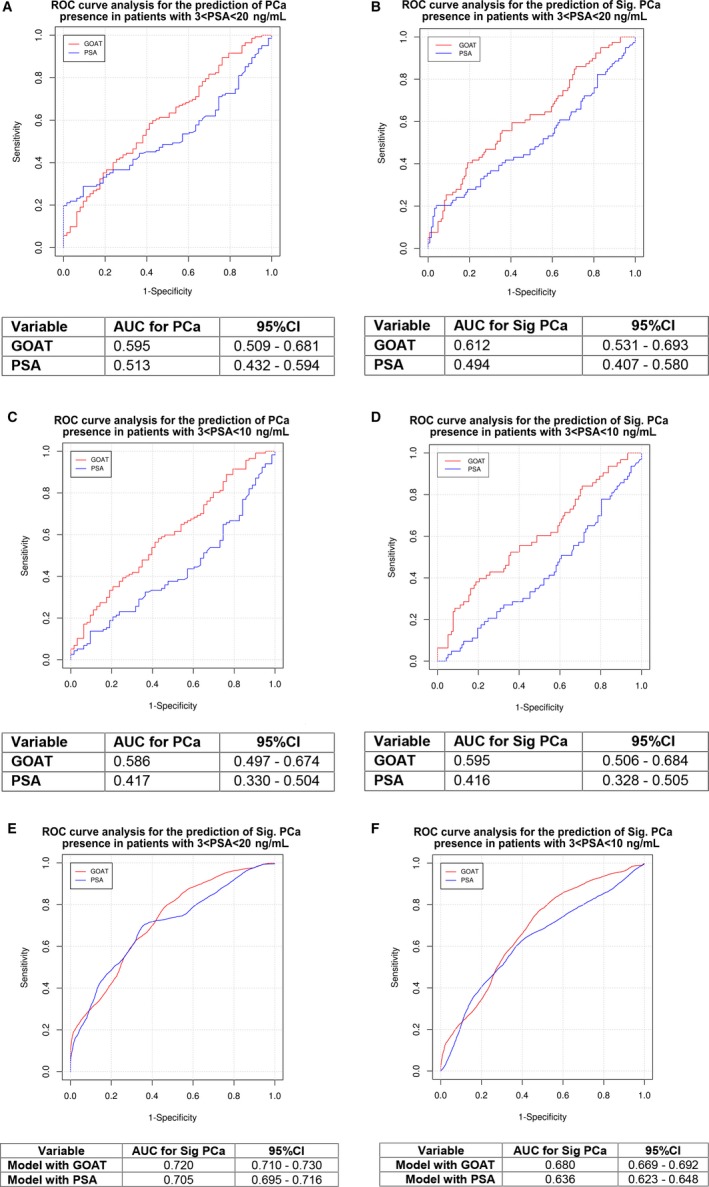
Capacity of plasma GOAT and PSA levels to predict the presence of prostate cancer (PCa) and significant (Sig) PCa. A‐D. Graphics showing the receiver operating characteristic (ROC) curves analyses of the capacity of GOAT (red line) and PSA (blue line) to diagnose: A, PCa in patients with PSA ranging 3‐20 ng/mL; B, Sig PCa in patients with PSA ranging 3‐20 ng/mL; C, PCa in patients with PSA ranging 3‐10 ng/mL; and D, Sig PCa in patients with PSA ranging 3‐10 ng/mL. E‐F, Graphics showing the ROC curve analysis of the capacity of models combining age, DRE and testosterone with GOAT levels (red line) or PSA (blue line) to predict the presence of Sig PCa in patients ranging 3‐20 ng/mL PSA levels (E), or in patients ranging 3‐10 ng/mL PSA levels (F). AUC and CI of each ROC curve are depicted in the tables below. These analyses were performed using patients with suspected PCa (cohorts 2 and 3)

To explore the potential capacity of prediction of plasma GOAT levels compared to PSA levels, patients from cohorts 2 and 3 with a PSA range between 3 and 20 ng/mL were analysed. This analysis revealed that GOAT was a better biomarker than PSA for the diagnoses of PCa [n = 140 PCa patients; GOAT levels: AUC = 0.595 (0.509‐0.681) vs PSA levels: AUC = 0.513 (0.432‐0.594); Figure 2A]. This difference between both biomarkers was particularly significant for the diagnosis of Sig PCa, wherein the AUC improved for GOAT levels and worsened for PSA levels, [n = 77 Sig PCa patients; GOAT: AUC = 0.612 (0.531‐0.693) vs PSA: AUC = 0.494 (0.407‐0.580); *P* = 0.035; Figure 2B].

This analysis was also applied to assess the predictive capacity of plasma GOAT levels, compared to PSA levels, in patients with a more restricted range of PSA, of 3‐10 ng/mL, the so‐called PSA grey zone (Figure 2C,D). The results clearly indicated that GOAT levels are a significantly better indicator than those of PSA to predict PCa in these patients [n = 117 PCa patients; GOAT levels: AUC = 0.586 (0.497‐0.674) vs PSA levels: AUC = 0.417 (0.330‐0.504), *P* < 0.01), Figure 2C]. Likewise, as illustrated in Figure 2D, the same was true for the population with Sig PCa, where GOAT levels significantly outperformed the predictive potential of PSA levels in this group of patients [n = 63 Sig PCa patients; GOAT levels: AUC = 0.595(0.506‐0.684) vs PSA levels: AUC = 0.416(0.328‐0.505); *P* < 0.01].

**Figure 3:**
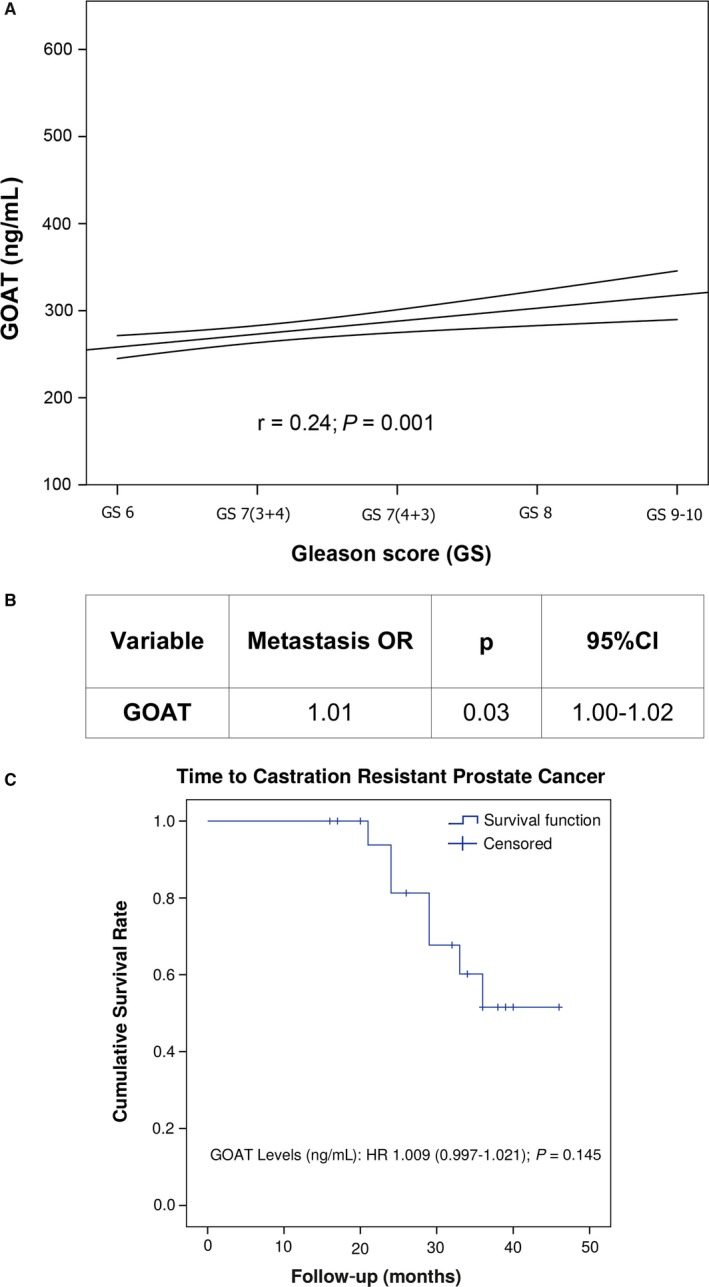
Association of plasma GOAT levels with aggressive features of prostate cancer (PCa) patients. A, Correlation between plasma GOAT levels and PCa Gleason Score. Coefficient of correlation was evaluated by Pearson's test. The graphic shows the lineal adjusted method and mean confidence interval. B, Association (odds ratio, OR) between plasma GOAT levels and the presence of metastasis at diagnosis evaluated by computerized tomography and bone scan. C, Representation of progression‐free survival curve from 19 patients treated with hormonotherapy. Results of univariate Cox regression analysis analysing the association of GOAT levels and the time to the event are depicted

Based on the previous results, a multivariate model based on GOAT or PSA levels complemented with an additional panel of clinical variables analysed in PCa (i.e. age, DRE and testosterone levels) was implemented to determine whether this combination could improve the accuracy of detection of PCa in patients with PSA levels between 3‐20 ng/mL (Figure 2E) and 3‐10 ng/mL (Figure 2F). This analysis revealed that the combination of this panel of clinical variables with plasma GOAT levels is significantly more efficient in detecting Sig PCa than when combined to plasma PSA levels [*P* < 0.001 in both cases; Figures 2E,F].

### 3.4 Association of plasma GOAT levels with aggressiveness features of PCa patients

Association between aggressiveness features of the cohort of patients with PCa revealed that plasma GOAT levels showed a significant correlation with GS (*r* = 0.24; *P* = 0.001; Figure 3A). Remarkably, high GOAT levels were associated with the presence of metastasis at the time of diagnosis, as evaluated by computerized tomography and bone scan (*P* = 0.03; Figure 3B). Furthermore, an exploratory analysis in the patients initially treated with hormonotherapy (n = 19) and a median follow‐up according to clinical practise of 35 months (26.75‐39) indicated a tendency in the association of plasma GOAT levels with an earlier castration‐resistant prostate cancer (CRPC) status (OR = 1.009: 95% CI (0.997‐1.021); *P* = 0.145; Figure 3C).

## 4 DISCUSSION

PCa is a major health problem and a leading cause of mortality and morbidity globally.^1^ PSA has been used as the gold standard biomarker for the diagnosis of PCa since the 1990s, although its use remains controversial because of its lack of specificity. Specifically, although the proportion of men with metastatic PCa at the time of diagnosis have decreased dramatically with the introduction of PSA as a screening test, more men are being diagnosed with PCa, with the majority having early stage, clinically indolent disease, the majority of which may never have led to harm.^12^ In addition, many men with benign conditions such as inflammation or hyperplasia are also being diagnosed and biopsied based on the results of the PSA test.^3^ Moreover, it has been proposed that treatment of indolent cancer may cause a patient more harm than good as biopsies and PCa treatments have been associated with psychological distress, loss of bodily function, pain, suffering for patients and with a decrease in the patient QoL.^13^ Consequently, these data have led to widespread criticism that PCa is now an “overdiagnosed” and “overtreated” cancer based on the PSA test. Therefore, there is an urgent need for the identification of new diagnostic and prognostic biomarkers for PCa, especially for Sig PCa, in order to improve the clinical management of PCa and to reduce the elevated number of biopsies and the overdiagnosis of non‐significant PCa.^4^

In this context, there have been numerous efforts to improve the performance of the PSA test based on PSA derivatives (ie, PSA “density,” PSA velocity and doubling time, free PSA, etc.); however, measurement of these derivatives has modestly improved care in that they are largely hindered by the same issues confounding PSA itself.^14^ Additionally, other non‐invasive biomarkers to diagnose PCa have been proposed [i.e. prostate cancer antigen 3 (PCA3), the gene fusion product TMPRSS2‐ERG, the 4k score test, the Prostate Health Index (PHI) in body fluids, multiparametric magnetic resonance imaging (mpMRI), etc.],^15^, ^16^, ^17^, ^18^, ^19^, ^20^ but many of these tests are currently adjunctive to PSA, and head‐to‐head studies to determine whether these tests perform well in the absence of PSA screening are lacking. Moreover, PSA remains an inexpensive test and, thus, costs and availability of these alternative tests minimize their implementation worldwide. Therefore, additional accessible biomarkers should be implemented in daily clinical practice, especially those with a prognostic and predictive value of Sig PCa at the point of screening, which is the current greatest unmet clinical need, as this may reduce unnecessary interventions.

In line with this, our group and others have recently demonstrated that GOAT enzyme is overexpressed (at the mRNA and/or protein level) in PCa tissues and PCa cell lines compared to healthy prostate tissues and normal cell lines,^10^, ^21^ and, most importantly, we also reported that GOAT is oversecreted in PCa cells compared to normal prostate cells.^10^ In fact, this initial, pilot study from our group revealed that plasma GOAT levels could discriminate between PCa and healthy subjects, suggesting that this enzyme might be used as a potential novel non‐invasive biomarker of PCa.^10^ However, this previous study was implemented with a limited cohort of patients and we could not establish therein whether plasma GOAT levels could be a better diagnostic marker than PSA for the diagnosis of PCa, specially on those individuals with PSA levels ranging between 3 and 20 ng/mL, the most ambiguous region wherein precision of PSA is remarkably poor, as well as for the diagnosis of Sig PCa. Consequently, the present study is the first to demonstrate that GOAT could be a significantly better diagnostic marker than PSA, exhibiting higher AUC, on those individuals with PSA levels ranging 3‐20 ng/mL and especially for the diagnosis of Sig PCa. In this scenario, it is worth noting that the overexpression of GOAT has been demonstrated at tissue level in other endocrine tumours,^22^ but, to the best of our knowledge, this is the first study evaluating GOAT plasma level and to analyse its putative utility as biomarker for cancer diagnosis. Therefore, although the role of GOAT as possible biomarker in other endocrine tumours cannot be completely ruled out and that its specificity for PCa needs to be further explored, this study strongly suggests that GOAT levels might represent a novel, valuable biomarker for Sig PCa.

We further explored the potential predictive capacity of plasma GOAT levels compared to PSA levels and found that plasma GOAT levels show a significant better AUC than plasma PSA levels in patients with PCa, but specially in patients with a PSA < 20 ng/mL (wherein the capacity of PSA is significantly worse) or most importantly, with a PSA < 10 ng/mL (known as the PSA grey zone), having an independent association with the clinical variables commonly used in clinical practice. Furthermore, a multivariate model based on GOAT or PSA levels complemented with an additional panel of clinical variables measured in PCa such as age, DRE and testosterone levels demonstrated that GOAT levels could be efficiently complemented with these clinical parameters to significantly increase its accuracy for the prediction of Sig PCa, which altogether reinforce the idea that GOAT enzyme might represent a promising biomarker, complementing PSA determination for the diagnosis of Sig PCa.

Based on the clear association found between plasma GOAT, but not PSA, levels with Sig PCa, we hypothesized that plasma GOAT levels in PCa patients might be linked to the aggressiveness of PCa. Remarkably, our results indicate that plasma GOAT levels could represent a novel predictive biomarker of aggressiveness, as we found that its levels are positively associated with GS (i.e., higher GOAT levels in patients with GS ≥ 7) as well as with the presence of metastasis at the time of diagnoses. Moreover, plasma GOAT levels tended to be associated with an earlier diagnosis of CRPC, which might also indicate that this enzyme may serve to develop future therapeutic target for PCa. In line with this, we have recently demonstrated that GOAT enzyme is positively correlated in PCa with the levels of the In1‐ghrelin splicing variant, but not with those of native‐ghrelin, wherein the presence of In1‐ghrelin variant drastically increased the aggressiveness features of PCa, acting as a true oncogene in this pathology.^23^ In fact, this previous study demonstrated that In1‐ghrelin silencing diminished the aggressiveness of PCa cells (e.g. proliferation capacity) suggesting that In1‐ghrelin could be considered as a novel target for the development of new and more specific therapies in PCa. When viewed as a whole, the results of the present manuscript indicating that GOAT levels are markedly elevated in Sig PCa and are associated to aggressiveness features in PCa (i.e. GS and presence of metastasis), together with the previous results showing a strong correlation of GOAT levels with In1‐ghrelin variant levels in PCa,^23^ invite to suggest that GOAT enzyme and In1‐ghrelin variants could be functionally linked in PCa, where In1‐ghrelin variant might be the primary target of GOAT, and that an autocrine/paracrine circuit involving these two components of the ghrelin system may possibly operate in PCa to increase the aggressiveness features of PCa cells, which set the stage for future investigations.

In sum, the present report provides the first comparative analysis to determine the potential utility of plasma levels of GOAT, in combination with other traditional clinical variables (i.e. age, DRE and/or testosterone), as diagnostic tools for the detection of PCa, using an ample cohort of patients (n = 312) and defining clinically relevant subgroups (e.g. Sig PCa vs non‐Sig PCa). Our results show, for the first time, that the measurement of plasma GOAT levels might represent a significantly better diagnostic marker than plasma PSA levels, exhibiting higher AUC, particularly on those individuals with PSA levels ranging 3‐10 ng/mL (the PSA grey‐zone) or 3‐20 ng/mL. Moreover, as plasma GOAT levels showed a significant better AUC than plasma PSA levels for the detection of Sig PCa and its levels were associated with aggressiveness features of PCa, we propose that the measurement of plasma GOAT levels, in combination with PSA and/or an additional panel of clinical variables measured in PCa (i.e. age, DRE and testosterone levels), might be considered as a novel, complementary, non‐invasive tool to provide a better diagnosis of PCa, especially for Sig PCa and for patients with grey‐zone PSA levels, as well as a putative tool for the prediction of PCa aggressiveness.

## AUTHOR CONTRIBUTIONS

E. Gómez‐Gómez, JM Jimenez‐Vacas and RM. Luque conceived and designed the project; E. Gómez‐Gómez, JM. Jiménez‐Vacas, J. Carrasco‐Valiente, AM. Blanca‐Pedregosa, J. Valero‐Rosa, T. González‐Serrano, M.D. Gahete, MJ. Requena‐Tapia, and RM. Luque acquired data; E. Gómez‐Gómez, JM. Jiménez‐Vacas, V. Herrero‐Aguayo, J. Carrasco‐Valiente, A. León‐González, JL. Fernández‐Rueda, MD. Gahete, MJ. Requena‐Tapia, JP. Castaño, and RM. Luque performed the analysis and interpretation of data; E. Gómez‐Gómez, JM. Jimenez‐Vacas and RM. Luque wrote the manuscript; J. Carrasco‐Valiente, V. Herrero‐Aguayo, AM. Blanca Pedregosa, AJ. León‐González, J. Valero Rosa, JL. Fernández‐Rueda, T. González‐Serrano, J. López‐Miranda, MD. Gahete, JP. Castaño and MJ. Requena‐Tapia revised the manuscript for important intellectual content; E. Gómez‐Gómez, JM. Jimenez‐Vacas, JL. Fernández‐Rueda and RM. Luque performed the statistical analysis; RM. Luque and MJ. Requena‐Tapia obtained funding; RM. Luque supervised the work.

RM. Luque is the guarantor of this work and, as such, had full access to all the data in the study and takes responsibility for the integrity of the data and the accuracy of the data analysis.

## CONFLICTS OF INTEREST

The authors confirm that there are no conflicts of interest and have nothing to disclose.
